# Case report of non-gene editing CD7 CAR T cell therapy in CD7^+^ Sézary syndrome: preclinical validation and first-in-human use

**DOI:** 10.3389/fimmu.2025.1604490

**Published:** 2025-08-01

**Authors:** Haichan Xu, Lihua Sun, Zehua Wu, Vincent M. DeStefano, Masayuki Wada, Jennifer E. Chow, Hui Yi, Guoling Wang, Jing Dai, Wei Zheng, Ting Wang, Wenli Zhang, Chengxing Song, Jing Luo, Yu Ma, Benjamin Waner, Mengjie Dong, Haibo Chen, Baozhen Qin, Hongyu Zhang, Jamie Hsing-Ming Chang, Yupo Ma, Jia Feng

**Affiliations:** ^1^ Department of Hematology, Peking University Shenzhen Hospital, Shenzhen, China; ^2^ Research & Development Division, iCell Gene Therapeutics Inc., Long Island High Technology Incubator, Stony Brook, NY, United States; ^3^ College of Medicine, Shenzhen University, Shenzhen, China; ^4^ Department of Hematology, Peking University Shenzhen Hospital, Shenzhen Peking University-The Hong Kong University of Science and Technology Medical Center, Guangdong, Shenzhen, China; ^5^ Department of Cell Therapy Center, Shenzhen Peking University-The Hong Kong University of Science and Technology Medical Center, Shenzhen, China; ^6^ Division of Manufacturing, iCAR Bio Therapeutics Ltd, Zhongshan, China; ^7^ Department of Nuclear Medicine, Peking University Shenzhen Hospital, Shenzhen, China

**Keywords:** Sézary syndrome, T cell malignancy, CAR T cells, CD7 CAR, immunotherapy

## Abstract

**Introduction:**

Sézary syndrome (SS) is a leukemic form of cutaneous T cell lymphoma (CTCL), distinguished from mycosis fungoides by the presence of cancerous lymphocytes in the blood and often bears very poor prognoses. SS treatment is palliative, and thus novel therapies are needed. The CD7 surface antigen is highly expressed and confined to the surface of T cells, therefore when present, serves as a promising target for immunotherapy.

**Methods:**

Herein we describe the preclinical validation and clinical application of our non-gene editing CD7 targeted chimeric antigen receptor (CAR) T therapy to treat relapsed/refractory (r/r) CD7 expressing SS. The CD7 CAR construct possesses a “safety switch” (RTX) to enable rapid depletion of the CAR T treatment with administration of rituximab. Preclinical evaluation of the CD7-RTX CAR T cells demonstrated >99% depletion of target cells in both co-cultures, at 1:1 and 2:1 effector: target (E:T) ratios, and mouse models. In a mouse model, “safety switch” testing resulted in rapid elimination of CAR T cells with rituximab infusion. RTX, in our CD7 therapy, has not yet been clinically validated.

**Results:**

A 53-year-old male diagnosed with r/r SS, expressing CD7, was treated with 2×10^6^ CD7-RTX CAR T cells/kg of body weight, as compassionate use. The patient achieved medication and symptom free complete remission (CR) within 28 days post-CAR. The patient remained in CR at 18-month follow-up. The treatment was well tolerated and without severe adverse events (SAEs).

**Discussion:**

Our CD7-RTX CAR T therapy demonstrates exceptional safety and efficacy in one patient with CD7^+^ r/r SS. This was the first recorded use of CD7 targeted CAR T therapy to treat SS. SS is prototypically CD7^-^, thus despite its efficacy in this patient, this treatment approach is likely not generalizable to most SS patients. However, this study supports the importance of thorough tumor characterization and the potential use of CD7-RTX CAR T cells to treat a variety of malignancies expressing CD7. Future clinical trials are required to characterize the safety and efficacy of CD7-RTX CAR T cells.

## Graphical Abstract

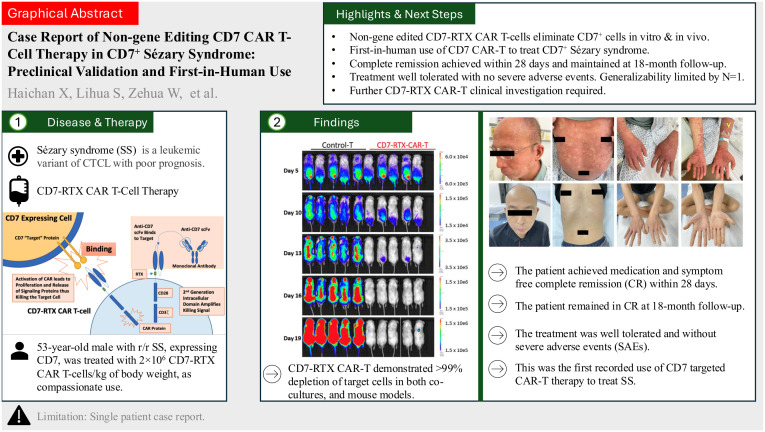

## Introduction

T cell malignancies, comprised of T cell leukemias and lymphomas, are heterogeneous hematological cancers. They originate from T cells at various developmental stages and often bear poor prognoses (1, 2). Cutaneous T cell lymphomas (CTCLs) are non-Hodgkin lymphomas (NHLs) primarily composed of Mycosis Fungoides (MF) and Sézary syndrome (SS) (3, 4).

SS, a leukemic variant of CTCL, is an aggressive disease which presents with diffuse infiltrating cutaneous lesions, erythroderma, non-scarring alopecia, palmoplantar keratoderma, systemic lymphadenopathy, and pruritus. SS commonly affects elderly patients with a median age of diagnosis between 60 and 65 years and bears a poor prognosis (5).

In a 12-year retrospective review of a multi-ethnic Caucasian and Asian cohort, Lim et al. reported 43% mortality in SS patients with a mean overall survival (OS) of 3.3 years from time of diagnosis (6). Similarly, Kubica et al. reported a 42% mortality at 5 years in SS patients and a 4-year median OS (7). The treatment for SS is systemic and tailored to the stage of the disease. Skin-directed electron beam, phototherapy, topical therapy, radiation, targeted agents, and chemotherapeutics have been trialed, however, relapse rates remain high (8–13). CTCLs such as MF and SS have no known cure and hence current treatment approaches are palliative (14).

CTCLs and more broadly, T cell malignancies require novel solutions that precisely and potently target the “root cause” of the disease. Notably, Vieyra-Garcia et al. suggest that CTCL inflammation results from recruitment and activation of benign T cells by c-Kit^+^OX40L^+^CD40L^+^ dendritic cells contributing to disease progression; targeting c-Kit, OX40, and CD40 signaling may offer new therapeutic approaches to treat MF (15). Additionally, chimeric antigen receptor (CAR) T cell technology may be implemented to treat T cell malignancies, and more specifically CTCLs, given its ability to expand *in vivo* and direct MHC-independent cytotoxicity to target cells. Six FDA-approved chimeric antigen receptor (CAR) T cell therapies currently exist to target B cell and plasma cell malignancies that express the CD19 and BCMA surface antigens respectively (16, 17). Given the success of FDA-approved CAR T therapies, this approach has the potential to achieve long-term symptom and medication-free complete remission (CR) in T cell malignancies.

The CD7 surface antigen is a transmembrane glycoprotein expressed on the majority of T cells and natural killer (NK) cells and is present on approximately 95% of lymphoblastic leukemias and lymphomas (18, 19). SS, however, is immunophenotypically CD7^-^ (20). Specifically, at least 90% of T cells are typically CD7^-^ on immunohistochemistry of MF in patch stage, with a specificity of 93% (21). In peripheral blood, loss of CD26 (≥80% CD4^+^ T cells) and/or CD7 (≥40% CD4^+^ T cells) alongside other gene expression biomarkers such as STAT4, TWIST1, DNM3, and PLS3 aid in diagnosis of 83% and 98% of SS respectively (22).

Currently, there exists no FDA approved CAR T cell therapy for the treatment of CD7-positive (CD7^+^) malignancies. This study details our novel non-gene edited, CD7 targeted, CAR T cell therapy expressing CD20 mimotopes (RTX) to allow for rapid depletion of the CAR T cells with rituximab infusion. In principle, the RTX functions as a “safety switch” should the patient decompensate. The non-gene edited approach promotes internalization of the CD7 surface antigen on the engineered cell to avoid fratricide.

We report the validation of our CD7-RTX CAR T cell therapy *in vitro*, in mouse models, as well as in a patient suffering from relapsed/refractory (r/r) SS expressing CD7, treated under compassionate use. This is the first-in-human use of CD7 targeted CAR T therapy to treat SS. Patient history, symptom progression, pre-/post- serum analysis, and imaging are included.

## Materials and methods

### Blood donors and tumor cell line

Peripheral blood mononuclear cells (PBMCs) were obtained from healthy patient samples in accordance with our Stony Brook University Institutional Review Board (IRB) approved protocol. All donors provided written informed consent. The CCRF-CEM cell line was acquired from ATCC (Manassas VA). T cells and CCRF-CEM cells were cultured as previously described (23, 24).

### Lentiviral vector production

The lentiviral vector was generated as previously described (23, 25–27). HEK293T cells were cultured in T flasks to achieve approximately 70-80% confluence. Next, cells were transfected with the CD7-RTX CAR containing expression plasmid and viral packaging plasmid as previously described (23). Incubation was performed as previously described (23). Following incubation, the resulting supernatant was harvested, filtered through a 0.2 µM disc filter, and stored at -80°C until utilized.

### Generation and characterization of CD7-RTX CAR T cells

The activation and transduction procedures of T cells utilizing lentiviral vector was described previously (23). CD7-RTX CAR T cell expression was analyzed using flow cytometry to detect F(ab)’ fragment, following previously described protocol (23). Flow cytometry was conducted using an FACS Calibur instrument (Becton Dickinson, Franklin Lakes, NJ), and results were analyzed using Kaluza software (Beckman Coulter, CA).

### Co-culture ablation assays

CD7-RTX CAR T cells or control T cells were incubated with CD7 expressing CCRF-CEM cells in 18 hour 1:1 or 2:1 co-culture experiments in 1 mL of T cell growth media, excluding IL-2, as previously described (23). Following 18 hours of co-culture, cells were stained with mouse anti-human CD3 (Cytek Tonbo) and CD7 (BD Biosciences) and then analyzed by flow cytometry.

### *In vivo* mouse model

*In vivo* activity of the CD7-RTX CAR T cell therapy was assessed in NOD scid gamma (NSG) mice that were sub-lethally irradiated and intravenously injected with luciferase-expressing CCRF-CEM cells. This study used all male mice, 8 to 12 weeks of age, with an approximate weight range of 25-30 grams obtained from Jackson Laboratories (Bar Harbor, ME, USA), strain (NOD.Cg-PrkdcsidIl2rgtm1Wjl/SzJ). All animal experimentation was performed in accordance with Stony Brook University’s Department of Laboratory Resources (DLAR), under an approved IACUC protocol in accordance with good laboratory practice (GLP) guidelines.

### Depletion of CD7-RTX CAR T cells with rituximab

Depletion of the CD7-RTX CAR T therapy through infusion of rituximab was verified *in vivo*. Each mouse was infused with 10×10^6^ CD7-RTX CAR T cells (Day 1) and subsequently injected with 150 µg (5 mg/kg) of rituximab on Days 5, 6, 7, 9 and 13. On Day 15, all mice were sacrificed for whole blood analysis. Blood samples were labeled using CD3, CD45, CD34 and F(ab)’ antibodies.

### GMP manufacturing of CAR T cells

The manufacturing of CAR T cells was carried out in a good manufacturing practices (GMP) laboratory. Peripheral blood apheresis PBMCs were obtained from allogeneic donors (the patient’s son, HLA 6/10 matched). After isolation of PBMCs, T cells were activated and transduced with lentiviral vector CD7-RTX for 48 hours. Subsequently, CAR cells were cultured and then tested for the presence of pathogenic microorganisms and contaminants. The CAR T cells were labeled and analyzed by flow cytometry to determine transduction efficiency.

### Patient information

The patient provided written informed consent for publication. A 53-year-old male first presented to the hospital in February 2018 with an erythematous, pruritic, disseminated rash over the total body surface. The patient was initially treated for allergic dermatitis, however, his condition progressed. In April 2019, biopsies of the left axillary lymph node and skin as well as peripheral blood analysis confirmed the diagnosis of SS with peripheral blood, bone marrow and lymph node involvement. With reference to the International Society for Cutaneous Lymphomas and European Organization of Research and Treatment of Cancer (ISCL/EORTC) staging system, the disease was categorized as T2N3B1M0 IVA-2 (28). After 1.5 years of palliative care, the malignancy progressed with symptomatic worsening of skin and lymph node disease. In May 2021, routine blood testing demonstrated significant leukocytosis: white blood cells (WBC) 41×10^9^/L, neutrophils (NEU) 11×10^9^/L (additional values: hemoglobin (HGB) 130g/L, platelets (PLT) 455x10^9^/L). Peripheral blood and bone marrow morphology indicated abnormal T lymphocytes. The bone marrow contained 53.76% of nuclear cells and expressed surface antigens CD3, TCRα/β, CD7, CD2, CD4, CD45RO, and weakly expressed CD5. The following surface markers were not expressed: CD8, CD56, CD57, CD30, CD16, CD45RA, CD26, CD10, CD25, granzyme B, perforin, CDL161, CD94, CD38, and TCRγ/δ. Bone marrow biopsy demonstrated clonal T cell proliferation, bone marrow invasion, as well as positive TCRγ and TCRβ rearrangement, negative TCD rearrangement, and *ATM* gene negative. The patient was assessed for lymphoma associated gene mutations which yielded positive results for *TET2, BCORL1, KMT2B, PLCG2, TYK2* mutations and *TP53* gene deletion. The skin pathological T cell phenotype was CD3(+), CD5(+), CD7(+), CD4(+, partial), CD8(-), PD-1(-). From May to October 2021, 8 courses of GVD (Gemcitabine, Vinorelbine, and Pegylated Liposomal Doxorubicin) and Dexamethasone were administered (first line of therapy), which led to symptom improvement, rash reduction by 60%, and lymph node size reduction by 50% indicating partial response (PR). Stable disease was confirmed by peripheral blood testing. Complications were observed following first-line treatment, notably, pulmonary bacterial and fungal infections, sacral and caudal skin infections (roseomonas mucosa and staphylococcus lugdunensis), as well as deep vein thrombosis. In November 2021, the patient returned with worsening lymph node enlargement, in addition to overall disease progression. Three courses of Brentuximab Vedotin, Chidamide (a HDACi) and thalidomide chemotherapy were administered as second line therapy, which led to stabilization of the disease. In February 2022, 3 courses of Mitoxantrone Liposomes combined with Selinexor were administered as third line therapy, which also resulted in disease stabilization (SD). However, severe allergic reaction appeared after the administration of Selinexor. In June 2022, a skin biopsy taken from the abdomen and the thigh indicated 10% tumor cell volume with a high tumor burden. After which, the patient was administered Venetoclax and Homoharringtine (HHT, an alkaloid derived from trees of the genus Cephalotaxus), Decitabine and Chidamide, Thalidomide, Methotrexate, and Interferon as fourth line therapy, and subsequently Camrelizumab (a PD-1 inhibitor) as fifth line therapy. The patient’s condition proved to be refractory to several lines of therapy consistent with disease relapse. No therapy trialed achieved and maintained complete response (CR). Given the patient’s severe r/r disease and CD7 surface antigen expression, the patient became eligible for treatment with our CD7-RTX CAR T therapy.

## Results

### Generation of CD7-RTX CAR T cells

**Figure 1:**
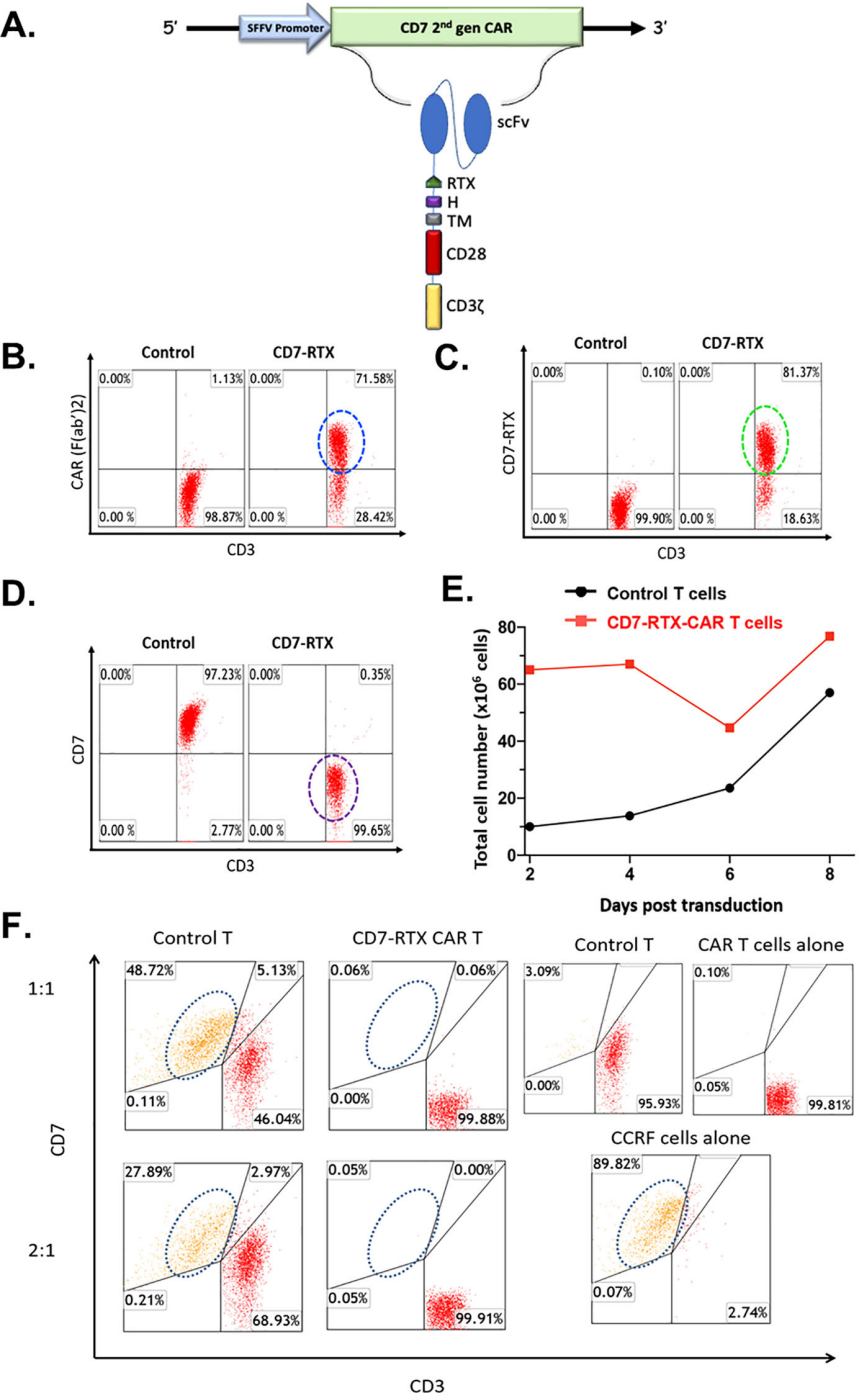
**(A)** CD7-RTX lentiviral vector schematic. Expression of the CD7-RTX CAR **(B)** Staining with goat-anti-mouse F(Ab’)2-Pe revealed a CAR expression of approximately 72%. **(C)** Staining with anti-human CD34 (to detect the RTX-binding epitope) demonstrated a transduction efficiency of approximately 81%. **(D)** Staining with anti-human CD3 and anti-human CD7 demonstrated CD7-RTX CAR T cells retained CD3 expression, however, lost expression of CD7. **(E)** CD7-RTX CAR T cells expanded at similar rates in comparison to control T cells, despite losing CD7 expression. **(F)** CD7-RTX CAR T cells demonstrated potent cytotoxicity against CCRF-CEM *in vitro*. Approximately 90% of CCRF-CEM cells were confirmed to express CD7 (bottom right panel). Control T cells (left panels) or CD7-RTX T cells (right panels) were incubated with CCRF-CEM cells in an 18-h co-culture at E:T ratios of 1:1 (first row), or 2:1 (second row). Target CD7 expressing cells are circled in each panel. Co-culture experiments demonstrated that CD7-RTX CAR T cells lyse 99.88% and 99.82% of the CCRF-CEM cells relative to control at 1:1 and 2:1, respectively.

The expression of our second generation CD7-RTX CAR T therapy is driven by a spleen focus-forming virus (SFFV) promoter. The extracellular CAR components are comprised of an anti-CD7 single-chain variable fragment (scFv), CD8-derived hinge (H) and transmembrane (TM) domains as well as a “safety switch” RTX domain. The intracellular domains consist of CD28 and CD3ζ signaling moieties (**Figure 1A**).

The CD7-RTX CAR T therapy is engineered through a single step, non-gene editing approach whereby the CAR binds to and internalizes the CD7 surface antigen, thus avoiding fratricide. Flow cytometry analysis demonstrated approximately 72-81% of T cells expressed the CD7-RTX CAR and that greater than 99% of T cells did not express CD7 (**Figures 1B–D**). Despite the loss of CD7 surface expression, CD7-RTX CAR T cells demonstrated comparable expansion to control T cells (**Figure 1E**).

### CD7-RTX CAR T cells specifically lyse CCRF-CEM cells *In vitro*

CD7-RTX T cells were co-cultured with CCRF-CEM cells and incubated for 18 hours. Potent lysis of CCRF-CEM cells was confirmed as the CD7-RTX CAR T cells achieved 99.88% and 99.82% depletion, relative to controls, at 1:1 and 2:1, respectively (**Figure 1F**).

### CD7-RTX CAR T cells exhibit remarkable anti-tumor cytotoxicity in a CCRF-CEM xenograft mouse model

To establish a xenograft mouse model, 12 NSG mice received a sub-lethal dose of gamma irradiation (2.0 Gy) and were assigned randomly to treatment or control groups. 1.0×10^6^ CCRF-CEM cells were infused 24 hours post-irradiation.

**Figure 2:**
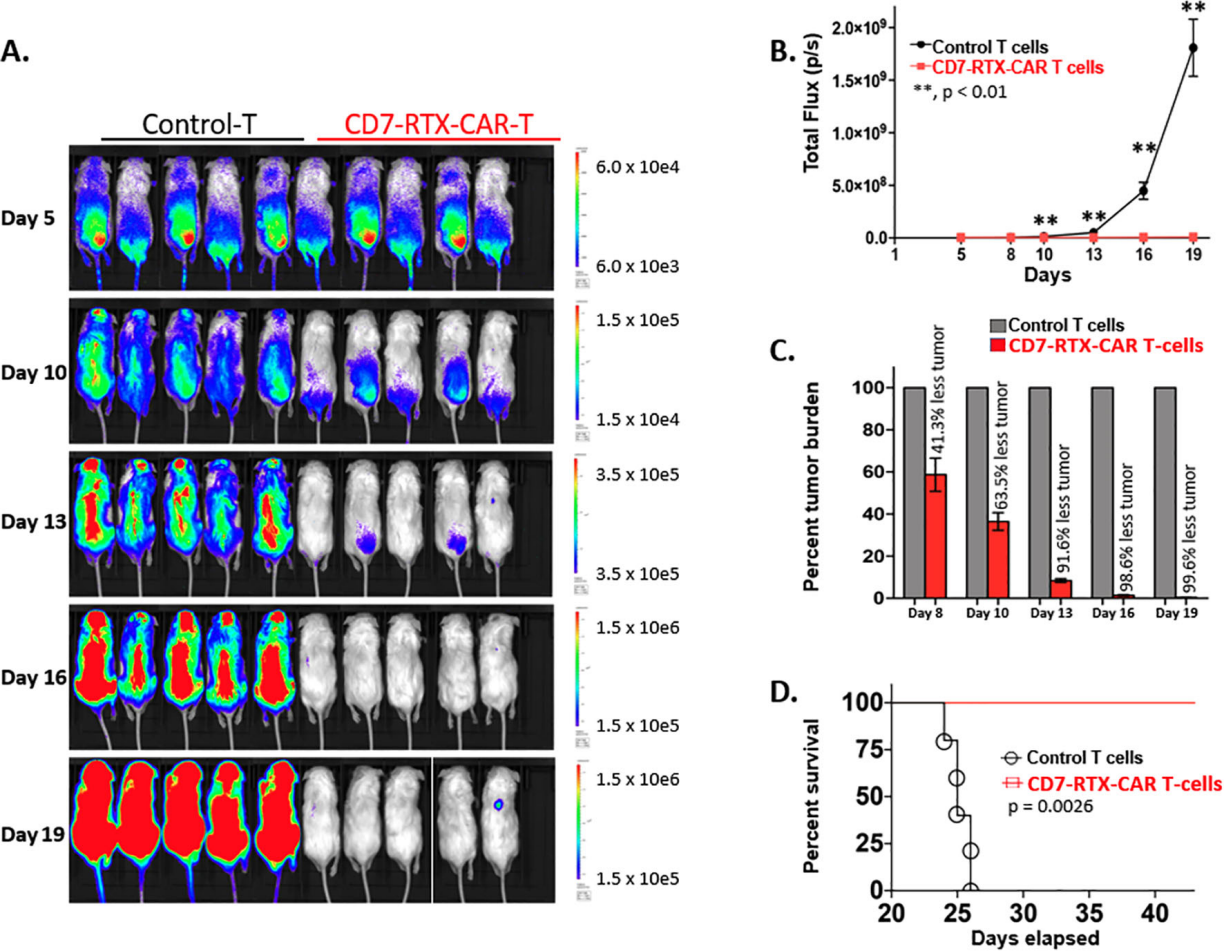
CD7-RTX CAR T cells demonstrate remarkable efficacy in a CCRF-CEM xenograft mouse model. **(A)** NSG mice were sub-lethally irradiated and then intravenously injected on day 1 with 1.0×10^6^ luciferase-expressing CCRF-CEM cells. Five days later, mice were injected with 10×10^6^ control or CD7-RTX CAR T cells. Mice were injected with RediJect D-Luciferin on days 5, 10, 13, 16, and 19 and then subjected to IVIS imaging, dorsal view. **(B)** Total flux (photons/second) was measured and indicated a significant difference (p < 0.01) in tumor burden between the two groups as early as day 8. **(C)** Percent tumor burden of CD7-RTX CAR T and control T cell-treated mice indicated a 99.6% reduction in tumor burden by day 19. **(D)** Kaplan-Meier survival curve of CD7-RTX CAR T versus control T cell-treated mice. Significant survival improvement was demonstrated in mice infused with CD7-RTX CAR T cells (p=0.0026).

Five days following introduction of CCRF-CEM cells, the mice were intravenously injected with 10×10^6^ CD7-RTX CAR or control T cells. To evaluate tumor burden on days 5, 10, 13, 16, and 19, RediJect D-Luciferin (Perkin-Elmer) was injected intraperitoneally and the mice were subjected to IVIS imaging to quantify luciferase activity. While flux, and thus tumor burden, continuously increased in control mice, it remained at approximately background levels for those treated with CD7-RTX CAR T cells (**Figures 2A, B**). While control mice were observed to have significant residual tumor burden in peripheral blood analysis, mice treated with CD7-RTX CAR T cells demonstrated remarkable >99% complete depletion (**Figures 2A, C**). Additionally, mice infused with the CD7-RTX CAR T therapy showed significant survival improvement (p=0.0026) in comparison to control T cell-treated mice (**Figure 2D**).

The CD7-RTX CAR T therapy demonstrated sustained absence of the CD7 surface antigen from T cells *in vivo*. Peripheral blood was extracted from the mice on day 20 and day 43, 15 days post-transduction and 38 days post-infusion, respectively, and evaluated by flow cytometry. Cells were first plotted against CD45 and side scatter (SSC). The CD45^+^ cells were then gated and analyzed for surface expression of CD3 and CD7 antigens. At day 20, CD7-RTX CAR T treated mice were observed to have approximately >94% CD3^+^/CD7^-^ T cells, demonstrating sustained downregulation of the CD7 surface antigen (**Supplementary Figure 1A**). By day 43, approximately >99% CD3^+^/CD7^-^ cells were observed (**Supplementary Figure 1B**).

The depletion of CD7-RTX CAR T cells through the infusion of rituximab was validated *in vivo*. Six NSG mice were sub-lethally irradiated and intravenously injected with 10×10^6^ CD7-RTX CAR T cells. Following CAR T cell expansion, on day 5, three mice were randomly injected with saline (control) or rituximab (150µg). Mice who received rituximab demonstrated approximately 96% reduction of CD7-RTX CAR T cells in peripheral blood as compared to controls (**Supplementary Figure 1C**).

### Clinical use of CD7-RTX CAR T cells to treat CD7+ Sézary syndrome

**Figure 3:**
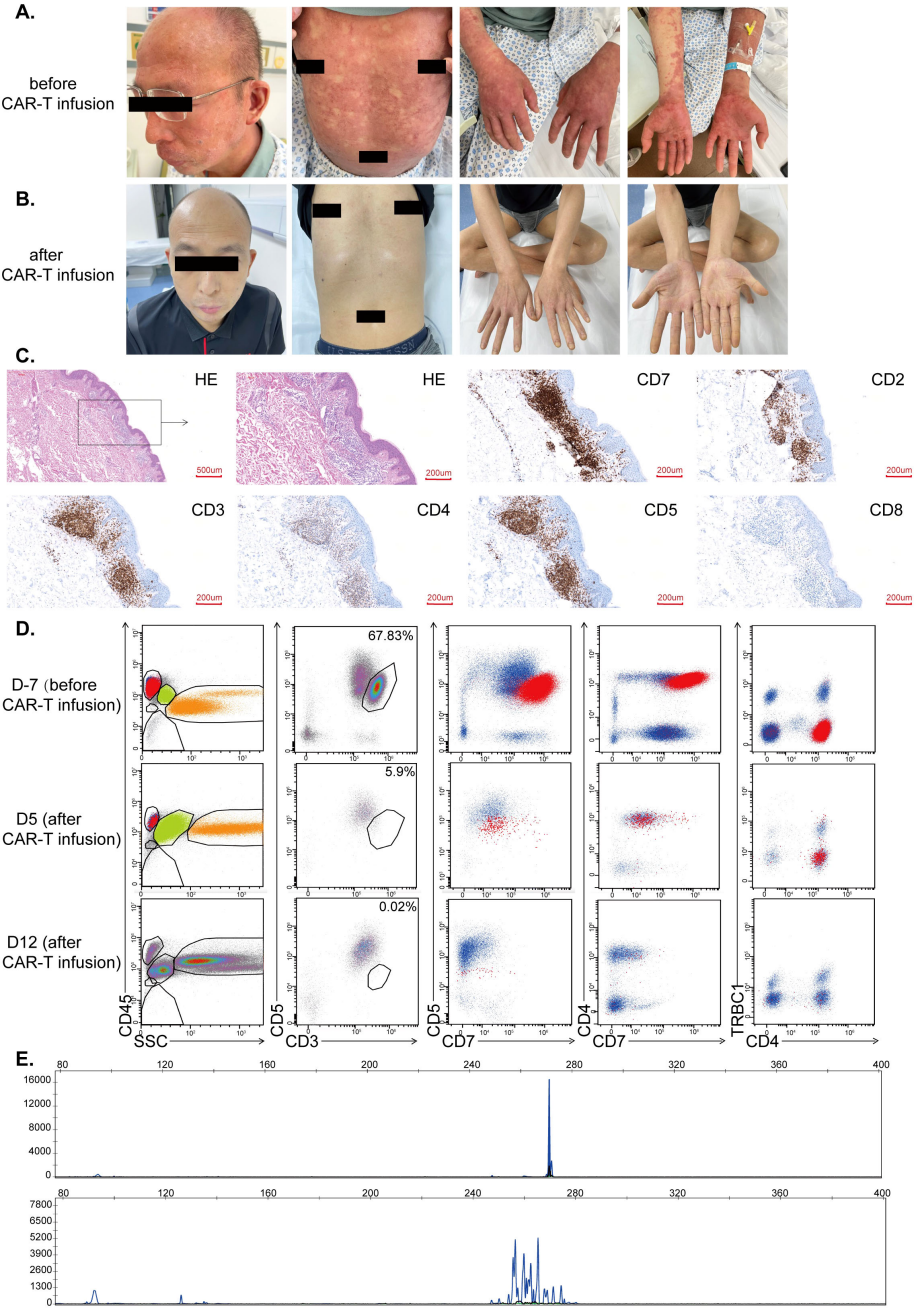
**(A)** Cutaneous disease prior to CD7-RTX CAR T treatment. Extensive lesions and erythroderma present. **(B)** Gross resolution of cutaneous disease, one-month post-CD7-RTX CAR T therapy. **(C)** The histopathological findings prior to CAR treatment demonstrated atypical lymphocytes that infiltrate the cutaneous and subcutaneous areas. These atypical cells stained positive for CD7, CD2 (partial), CD3, CD5, CD4 (partial) and negative for CD8 **(D)** Evaluation of peripheral blood 7 days prior to treatment demonstrated malignant T-lineage cells (upper panels, 67.83%) and 5-12 days post-treatment (middle panels, 5.9% and lower panel, 0.02% which is the background level). **(E)** TCR clone (TBB) distribution before treatment (upper panel) and 17 days after treatment (lower panel).

A patient suffering from r/r SS was treated as compassionate use with CD7-RTX CAR T cells. Prior to CAR T treatment, advanced disease status was confirmed in February 2023, characterized by lymph node infiltration, extensive cutaneous disease, and presence in peripheral blood. On physical examination, the patient displayed an extensive full body, pruritic, erythematous skin rash (**Figure 3A**). Before treatment, malignant T-lineage cells were quantified at 67.83% (**Figure 3D**, upper panels) by flow cytometry, and demonstrated a single peak, suggesting clonal origin, by T cell receptor (TCR) evaluation (**Figure 3D**, upper panel). Histopathological analysis demonstrated a substantial burden of CD7^+^ malignant cells within the skin prior to CAR-treatment (**Figure 3C**).

Fludarabine and cyclophosphamide were administered for conditioning prior to CD7-RTX CAR T cell therapy (February 3, 2023). The patient was infused with about 2×10^6^ CD7-RTX CAR T cells/kg body weight (February 8, 2023). Evaluation of peripheral blood one-week post-CD7-RTX CAR T treatment revealed partial remission (PR) with 97.29% reduction in tumor burden. Peripheral blood assessment at day 12 indicated >99% reduction in tumor burden with molecular complete remission (CR) achieved at day 28 post-treatment (**Figures 3D, E**), and resolution of cutaneous disease (**Figure 3B**). Day 28 post-CAR infusion, TCR morphology results indicated polyclonal T-lymphocyte presence with a greater than 3-fold reduction in maximum peak intensity (**Figure 3D**, bottom panel). No signs of graft versus host disease (GVHD) were found during the follow-up period.

**Figure 4:**
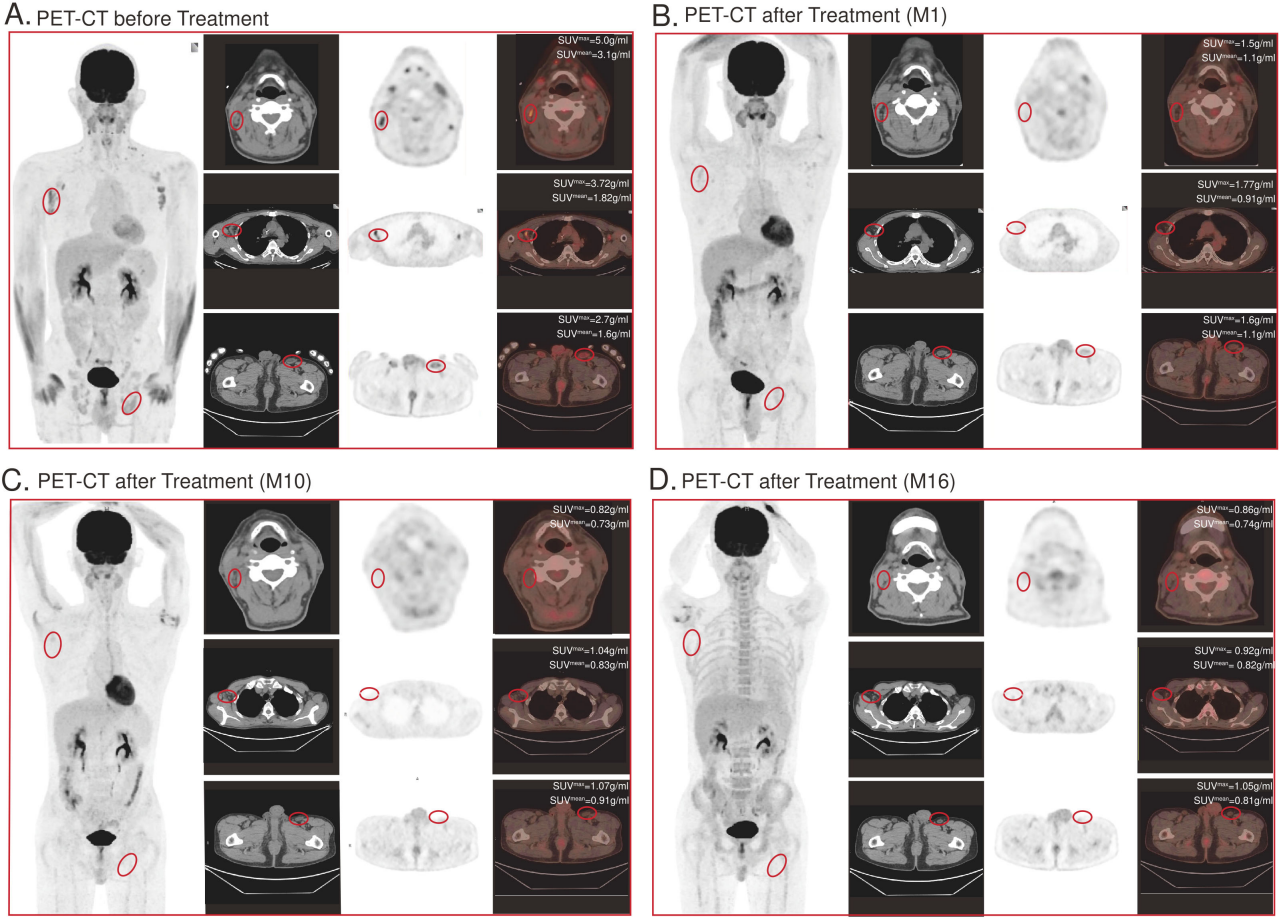
CD7-RTX CAR T cell treatment significantly reduces tumor burden. Top panel shows CT images, the middle panel shows the radionuclide distribution, and the bottom panel shows PET-CT fusion images. **(A)** PET-CT image demonstrates lesions of high uptake prior to treatment: (1) over the right temple (View 1), (2) right axilla (View 2), and (3) left inguinal region (View 3). **(B–D)** PET-CT image 1 month, 10 months, 16 months post-treatment. Absence of bulk disease appreciated: (1) over the right temple (View 1), (2) right axilla (View 2), and (3) left inguinal region (View 3).

Positron emission tomography-computed tomography (PET-CT) imaging was performed prior to treatment and demonstrated high uptake in lesions over the right temple, right axilla, and left inguinal region (**Figure 4A**). Repeat imaging at 1 month, 10 months, 16 months post-infusion demonstrated absence of signal suggesting resolution of disease (**Figures 4B–D**).

The patient underwent haploidentical allogeneic hematopoietic stem cell transplantation (the donor was his son, the same donor of CAR T cells) about one month after CD7-RTX CART treatment. The last follow-up date was September 2024, which was 18 months after bone marrow transplant, and the patient was in continuous CR.

**Figure 5:**
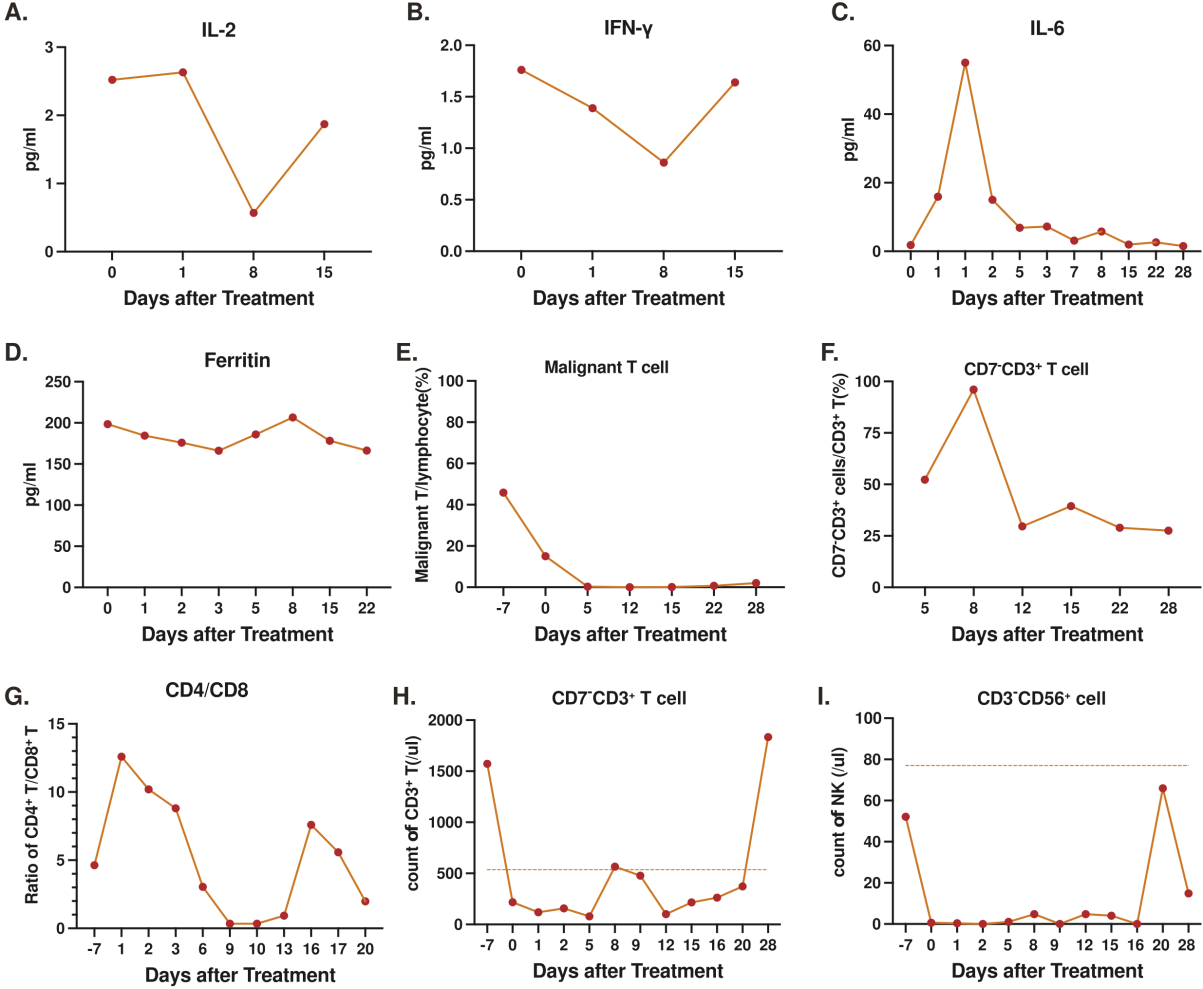
Levels of serum markers and cell counts, prior to and post-infusion of CD7-RTX CAR T cells: **(A)** interleukin (IL)-2 **(B)** interferon (IFN)-γ **(C)** IL-6 **(D)** Ferritin **(E)** Percent malignant T cells to total PBMCs **(F)** Percent CD7^-^ T cells to total T cells **(G)** Proportion of CD4^+^ to CD8^+^ T cells. **(H)** Count of T cells in peripheral blood (the normal range of T cells in peripheral blood is 536-1787 cells/µL) **(I)** Count of NK cells in peripheral blood (the normal range of NK cells in peripheral blood is 77-427 cells/µL).

Peripheral blood levels of interleukin (IL)-2 and interferon (INF)-γ demonstrated a decrease from baseline eight days (D8) following CD7-RTX CAR T treatment, and both returned to D0 levels, 15 days post-treatment (**Figures 5A, B**). Analysis of IL-6 indicated a rapid increase in the cytokine (50-60 pg/mL) approximately 1-2 days post-infusion which returned to near undetectable levels by D7 (**Figure 5C**). Ferritin levels remained near constant throughout, trending between 150-200 pg/mL from D0 to D22 (**Figure 5D**). The maximum cytokine levels and the time of peak (days post-CAR) can be found in **Supplementary Table 1**. Following infusion of CD7-RTX T cells the percentage of CD7^+^ malignant T cells diminished rapidly as compared to the total PBMC count (**Figure 5E**). Expansion of CD7 negative RTX CAR T cells was observed as levels increased from D5 (50%) to D8 (100%), before returning on D12 (25%) and remaining approximately constant to D28 (**Figure 5F**). CD8^+^ T cell expansion was observed in proportion to CD4^+^ T cells (**Figure 5G**), corresponding to the infusion and replication of CD7-RTX T cells on D8 (**Figure 5F**). On day 28, the patient’s peripheral blood T cell count had recovered to the normal range (1834 cells/µL), whereas the NK cell count, although gradually increasing to 65.97 cells/µL, remained below the normal threshold (77 cells/µL) and exhibited fluctuations. (**Figures 5H, I**).

Remarkable safety was demonstrated with no severe adverse events (SAEs). The patient was found to have pneumonia post-treatment, consistent prior pneumonia history, however this resolved with supportive care. Two days following CD7-RTX CAR T cell treatment the patient developed a fever of 39.2°C and tachycardia with a maximum heart rate of 120 beats per minute (BPM), both of which resolved with supportive care. No other adverse events (AEs) occurred.

## Discussion

The CD7-RTX CAR T treatment is engineered through a novel non-gene editing approach which allows for reduction in manufacturing steps. This also reduces the potential for off-target effects associated with gene editing. During the creation of our CD7-RTX CAR T therapy, the phenomenon of internalization of the CD7 surface antigen was an unexpected finding. CD7 is expressed on both normal and malignant T cells, posing a risk of significant fratricide in anti-CD7 CAR T therapy, a challenge overcome with our non-gene editing platform (19). The internalization process occurs during the manufacturing of our CAR T cell product, whereby binding of the CD7 surface antigen results in absence of the surface antigen on our therapy. However, when the CD7-RTX CAR T cells bind the target, an MHC-independent intracellular cascade occurs resulting in cytotoxic killing of the tumor cell.

The CD7-RTX CAR T therapy demonstrated remarkable >99% depletion of CCRF-CEM target cells in 18-hour co-culture experiments at 1:1 and 2:1 E:T ratios, confirming potent, on-target, cytotoxicity *in vitro*. *In vivo*, CD7-RTX CAR T cells achieved >99% killing of CCRF-CEM target cells and demonstrated expedient depletion of CAR T cells with rituximab infusion. The novel CD7-RTX CAR T treatment was validated clinically in a 53-year-old male patient diagnosed with r/r SS, expressing CD7. The patient was treated as compassionate use with 2×10^6^ CD7-RTX CAR T cells/kg of body weight and achieved CR within 28 days. The patient remained in CR at the 18-month follow-up. The treatment was well tolerated and without severe adverse events (SAEs).

Cytotoxic effector cells are critical to proper immune function, and depletion may be considered incompatible with life (29). CD7 is expressed by T cells, NK cells, and their precursors, therefore, infusion of the CD7-RTX T therapy rapidly depletes these vital subsets (19). Despite this potential for life-threatening T cell aplasia, our clinical data demonstrates expansion of CD7^-^ T cell populations. This observation is supported by Tan et al. who reported non-CAR T and NK cells were predominantly CD7^-^, and restored lymphocytes to normal levels in approximately half of participants, in a 2-year follow-up of r/r T-ALL patients treated with donor-derived CD7 CAR T cells (30). Similarly, Zhang et al. demonstrated that patients treated with anti-CD7 CAR T cell therapy experienced rapid expansion of CD7^-^/CD3^+^ lymphocytes sufficient to maintain effector adaptive immune function (31).

The CD7 surface antigen represents a favorable target to treat T cell malignancies since it is expressed in >95% of lymphoblastic T cell leukemias and lymphomas (32). CTCLs such as SS prototypically lose the expression of the CD7 surface antigen, however, our study reinforces the importance of thorough tumor characterization (20). Although many mature T cell lymphomas may not typically express the CD7 surface antigen, the exceptional safety and powerful efficacy demonstrated by our CD7-RTX CAR T therapy support its potential use and further investigation in tumors screened and found to have high homogenous CD7 expression, thereby advancing a personalized treatment approach.

The r/r SS patient treated with CD7-RTX CAR T therapy was found to have significant CD7^+^ disease burden and thus was successfully treated with our immunotherapy. The CD7 surface antigen is often lost or dim in SS, a phenomenon considered in SS diagnostic algorithms. Although SS is immunophenotypically CD7^-^, the patient treated in this study suffered from SS expressing this surface antigen. The atypical nature of this patients’ tumor profiling served as justification for compassionate use. Therefore, this case report is likely not generalizable in treatment of SS. While no data currently exists regarding the use of CD7-RTX CAR T therapy in other malignancies expressing CD7, proof of concept introduced in this case report suggests that use of CD7 directed immunotherapy may be an effective treatment approach and should be further explored. T cell leukemia (T-ALL) commonly expresses the CD7 surface antigen, but it may also be expressed in rare diseases such as, but not limited to, chronic lymphoproliferative disorders of NK cells (CLPD-NK), T cell prolymphocytic leukemia (T-PLL), T large granular lymphocytic leukemia (LGL) and enteropathy-associated T cell lymphoma (EATL) (19, 33–36). EATL is an aggressive, rare non-Hodgkin lymphoma that has a poor prognosis with an estimated median survival of 7.5 months and five-year survival of approximately 11-20%, thus, novel treatment approaches are desperately needed (37, 38).

This study validates the dosing of our CD7-RTX CAR T therapy. The excellent safety and efficacy demonstrated by CD7-RTX CAR T is consistent with the use of anti-CD7 CAR T treatments to date. No SAEs were recorded. The post-CAR pneumonia, fever of 39.2°C and tachycardia up to 120 BPM all resolved with supportive care, and no other AEs occurred. The safety profile observed in this case is encouraging, but a comprehensive assessment of clinical safety is limited given this was a single patient study. By day 28 post-CAR, T cell counts recovered to the normal range while NK cell counts continued to fluctuate below threshold. Despite lower than threshold NK cell counts, sufficient adaptive and innate immune function, is suggested by the absence of severe infection, including those in the skin to date. However, our data reinforces that CD7 is expressed on normal T and NK cells, thus, on-target off-tumor elimination of these adaptive and innate immune cells has the potential to induce immunodeficiency (39). In a single-center, phase I trial, Pan et al. report a high rate of 90% CR (n=18) and comparable safety profile for anti-CD7 CAR therapy to treat r/r T-ALL, with remission stable at median follow-up of 6.3 months (n=15) (40). Our CD7-RTX CAR T therapy possesses a “safety switch” in which the CAR therapy could be rapidly depleted with the infusion of rituximab, as validated in our mouse models. The utility of a “safety switch” to reduce CAR toxicity has been validated by Lin et al. who successfully aborted a CD5 targeted CAR treatment (expressing truncated epidermal growth factor (tEGFR)) with cetuximab infusion, in a patient observed to have dermatologic CAR related side effects (41). Similarly we have previously demonstrated the use of low dose CAMPATH (alemtuzumab) to bind to CD52, inducing cell death, and hence aborting CAR treatment *in vivo* (24, 42). Thus, inclusion of the RTX domain in our construct may feasibly serve as an additional safety measure as suggested by our mouse model, however this has not been validated in humans.

The preclinical and clinical use of the novel CD7-RTX CAR T therapy in a patient diagnosed with r/r SS demonstrated exceptional safety and efficacy achieving CR in 28 days with no SAEs and all AEs resolving with supportive care. This led to objective as well as symptomatic improvement of his aggressive disease. No signs of GVHD were found after CAR T infusion. This is comparable as GVHD was mild in other anti-CD7 CAR T studies (31, 43).

This case report is the first known use of CD7 targeted CAR to treat SS. Although CD7 expression is not inherently common in SS, future clinical trials will be designed to investigate the use of CD7-RTX CAR T therapy in diseases that do typically express CD7.

## Data Availability

The raw data supporting the conclusions of this article will be made available by the authors, without undue reservation.
