# Supplementary material for: Case report of non-gene editing CD7 CAR T cell therapy in CD7+ Sézary syndrome: preclinical validation and first-in-human use
Source: Front Immunol. 2025 Aug 1;16:1604490. doi: 10.3389/fimmu.2025.1604490 (PMC12354618; doi:10.3389/fimmu.2025.1604490)
Supplement: Supplementary file 1 [file Table1.docx]

**Validation of CD7 Targeted CAR T Cell Therapy and Clinical Use in CD7+ Sézary Syndrome**

**Supplemental Materials**


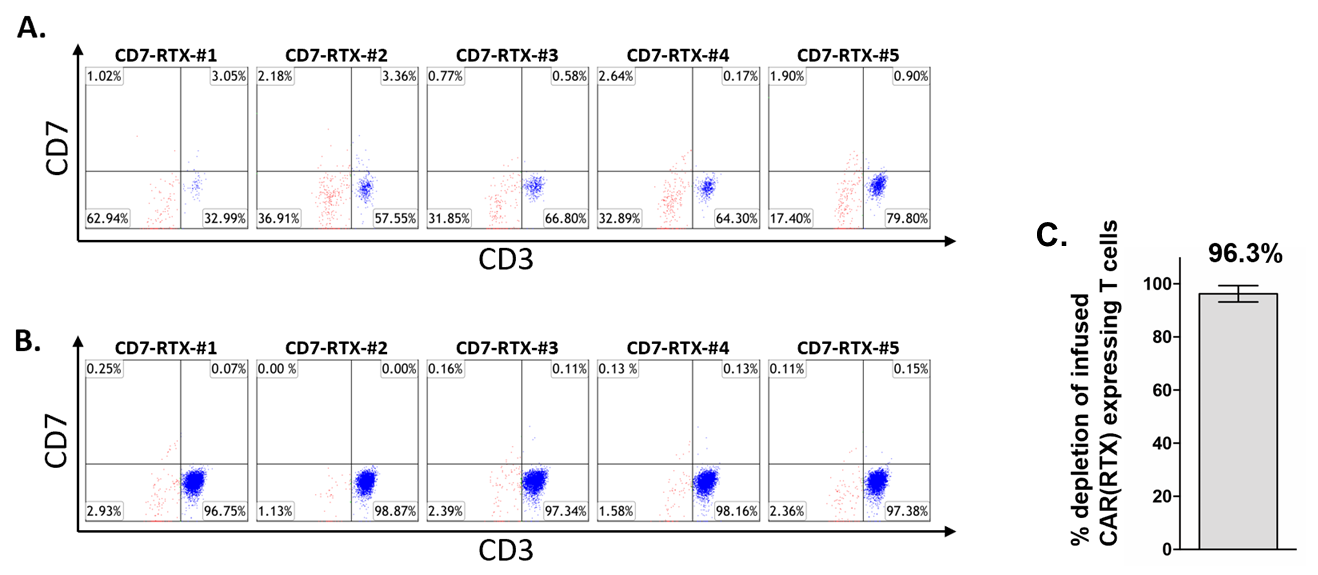


**Supplemental Figure 1.** (A) On Day 20 (15 days post-infusion of CAR T cells), blood was collected from the submandibular vein of each mouse to determine the persistence of infused CD7-RTX CAR T cells using flow cytometry analysis. CD7-RTX CAR T cell treated mice were observed to be CD3+/CD7- (blue dots). CD7-RTX CAR T cells retained CD7 suppression. (B) On Day 43 (38 days post-infusion of CAR T cells), blood was collected from the submandibular vein of each mouse to determine the persistence and confirm CD7 suppression of CD7-RTX CAR T cells using flow cytometry analysis. Samples were labeled using CD45, CD3 and CD7 antibodies. First, cells were gated by side scatter (SSC) and CD45 expression (data not shown). Then, mice treated with CD7-RTX CAR T cells showed CD3+/CD7- T cells (blue dots). This indicated CD7-RTX CAR T cells retained CD7 suppression on their surface for an extended duration. (C) Blood samples were labeled with CD45, CD3 and CD34 antibodies. Three doses of rituximab were administered, and cells were gated by SSC and CD45 expression. Then CD45+/CD3+ positive cells were further gated and plotted for CAR (RTX:CD34) and CD3 expression. Following five doses of rituximab, flow cytometry analysis revealed depletion of 96.3% of CD7-RTX CART cells.

**Table 1.** The maximum level of each respective cytokine detected in serum and the corresponding peak time.

| Cytokine | Level and Peak time (pg/mL) |
| --- | --- |
| TNFβ | 1.49 (D1) |
| IL-2 | 2.6 (D1) |
| IL-4 | 1.31 (D1) |
| IL-5 | 2.99 (D0) |
| IL-10 | 4.49 (D1) |
| TNFα | 4.22 (D1) |
| IL-1β | 2.13 (D1) |
| IL-8 | 6.87 (D0) |
| IL-15 | 11.77 (D8) |

**Table 2.** Adverse Reaction Record Form

| Adverse reactions and classification | Patient-QJX |
| --- | --- |
| CRS | Level 1 |
| Fever (body temperature > 38 °C) | 39.2 (D2) |
| low blood pressure |  |
| Hypoxemia |  |
| ICANS |  |
| Aphasia/confusion/cognitive impairment |  |
| epileptic seizure |  |
| motor dysfunction |  |
| Increased intracranial pressure or cerebral edema |  |
| hematological toxicity |  |
| anemia |  |
| neutropenia |  |
| Lymphopenia |  |
| Thrombocytopenia |  |
| CD4 cell deficiency** |  |
| Gastrointestinal reaction |  |
| Anorexia |  |
| nausea/vomiting/diarrhea |  |
| bloating |  |
| cardiovascular toxicity |  |
| chest pain |  |
| tachycardia | 120 BPM (D2) |
| heart failure |  |
| general |  |
| weak |  |
| flu-like symptoms |  |
| rash |  |
| Peripheral edema |  |
| lab test |  |
| Elevated AST |  |
| Elevated ALT |  |
| Elevated bilirubin |  |
| Increased creatinine |  |
| Elevated LDH |  |
| Infect |  |
| septicemia |  |
| lung infection | Yes, and there is a history of lung infection before reinfusion |
| intestinal infection |  |
| urinary tract infection |  |
| skin and soft tissue infection |  |
| oral infection |  |
| CMV reactivation |  |
| EBV reactivation |  |
| Coagulation disorders |  |

The grading of adverse reactions should be based on the latest version of CTCAE (Common Terminology Criteria for Adverse Events) (e.g. version 5.0). Since hematological toxicity is assessed for association with DLT, AE recovery time should be documented. Record the recovery time of all Level 3 and Level 4 AEs. *CD4 cell deficiency refers to CD4 cell absolute values of less than 200/ul.
